# Sustainable Protocols for Cellulose Nanocrystals Synthesis from Tomato Waste and Their Antimicrobial Properties against *Pseudomonas syringae* pv. *tomato*

**DOI:** 10.3390/plants12040939

**Published:** 2023-02-18

**Authors:** Daniele Schiavi, Riccardo Ronchetti, Veronica Di Lorenzo, Riccardo Vivani, Stefano Giovagnoli, Emidio Camaioni, Giorgio M. Balestra

**Affiliations:** 1 Department of Agriculture and Forest Sciences (DAFNE), University of Tuscia, Via San Camillo de Lellis snc, 01100 Viterbo, Italy; 2 Department of Pharmaceutical Sciences (DSF), University of Perugia, Via del Liceo 1, 06123 Perugia, Italy

**Keywords:** cellulose nanocrystals, tomato, enzymatic digestion, antimicrobial, bacteria, circular economy

## Abstract

Nanotechnology is rapidly gaining ground in crop protection, with the growing quest for sustainable nanopesticides and nanocarriers for plant pathogen management. Among them, cellulose nanocrystals (CNC) are emerging as innovative agrofood-waste-derived antimicrobial materials. In this work, new chemical and enzymatic CNC extraction methods from tomato harvest residues were evaluated. The obtained nanomaterials were characterized and tested for their antimicrobial properties on *Pseudomonas syringae* pv. *tomato* (Pto), the causal agent of bacterial speck disease on tomato. Both protocols were efficient. The enzymatic extraction method was greener, producing purer CNC at slightly lower yield. The obtained CNC, although they weakly inhibited cell growth and did not promote reactive oxygen species (ROS) formation, provoked bacterial aggregation and the inhibition of biofilm production and swimming motility. Both protocols produced CNC with similar morpho-chemical features, as well as promising antimicrobial activity against plant bacterial pathogens, suggesting their potential role in sustainable crop protection strategies. The new protocols could be a valuable alternative to conventional methods.

## 1. Introduction

Cellulose is one of the most versatile and abundant natural polymers on Earth. Cellulose is the main component of the plant cell wall, and it derives from repeated units of cellobiose, a glucose dimer, arranging themselves in long fibrils that present crystalline and amorphous regions [1]. Cellulose’s extraordinary mechanical properties have been well known since ancient times, but only in the last few decades has its exploitation in the form of nanomaterials been investigated. Through different processes, cellulose can be reduced to nanofibrils and nanocrystals. Cellulose nanocrystals (CNC) are acicular particles ranging from 50 to 500 nm in length. Owing to their high stiffness, low density and thermal expansion coefficient, high elastic modulus, and the abundant presence of surface hydroxyls groups, CNC have been employed in a broad range of applications, from reinforcement phase in packaging to nanocarriers for the delivery of active compounds [2]. Furthermore, CNC are biocompatible and non-toxic, suggesting their use in the biomedical and agrifood sectors [3,4]. As a result, cellulose and CNC markets have been predicted to expand globally in the next years. This is also evidenced by the increasing number of scientific works published in the last decade [5]. Despite the numerous innovative applications, CNC supply still remains one of the major issues in terms of sustainability. Two major approaches have been proposed over the years: finding new sources of cellulose and designing new protocols for cellulose extraction and CNC synthesis [6,7]. Alternative sources to commercial cellulose powder have been evaluated, including sunflower and sugarcane stalks, kiwifruit pruning residues, walnut shells, hemp fibers, and rice husks [8,9,10,11]. Indeed, the exploitation of agricultural waste is a tremendous added value for farmers and the whole production chain. Among the extraction and synthesis protocols, chemical bleaching and TEMPO-mediated oxidation, followed by acid hydrolysis, are the most common used with raw biomasses. In addition, mechanical defibrillation, acidic deep eutectic solvents, enzymatic digestions, and their combination have been proposed in an attempt to lower the amount of chemicals employed without altering CNC properties [12,13,14]. In the last decade, the investigation of microbial-assisted digestion for CNC extraction has paved the way for the use of enzymes [15]. Siqueira et al. (2010) explored the effects of exo- and endoglucanase on nanocellulose coming from sisal fibers, while Li et al. (2016) optimized a protocol for extracting cellulose from wheat straw by adding a xylanase digestion step [16,17].

In line with these studies, this work aims to explore the possibility of extracting CNC from tomato harvesting waste by applying chemical and enzymatic protocols, granting the preservation of CNC properties. Tomato is one of the most cropped species globally, playing a crucial role in many agricultural and food processing industries, especially in the Mediterranean region [18,19]. Although numerous approaches have been applied to extract CNC from tomato residues, such as stems and peels, none of them have employed enzymatic approaches [20,21]. To the best of our knowledge, this is the first work proposing the introduction of an enzymatic step in CNC extraction from tomato wastes. Moreover, due to CNC behavior and effects in a biological environment having been poorly characterized, the obtained CNC were tested against bacterial cells belonging to the model plant pathogen *Pseudomonas syringae* pv. *tomato* (Pto), the causal agent of bacterial speck disease on tomato [22,23]. Our results suggest that the addition of enzymatic protocols could enhance the sustainability of extraction procedures. The obtained CNC can significantly interfere with bacterial–environmental interaction processes. These observations strongly support the role of CNC as novel nanoagrochemical in sustainable crop protection strategies, contributing at the same time to the circular economy value of tomato waste-derived products [24,25].

## 2. Results

### 2.1. Cellulose Extraction and CNC Synthesis from Tomato Waste

Both of the proposed protocols to isolate cellulose from tomato waste gave good results. In particular, the chemical protocol extraction yield was 28.8% ± 1.2, while in the modified protocol, the extraction yields before and after enzymatic digestion were 28% ± 0.8 and 13.3% ± 0.9, respectively. CNC were successfully synthesized from both the chemically (CNC-CH) and the enzymatically (CNC-EN) extracted cellulose, with average hydrolysis yields of 21.6% ± 3.2 and 31.3% ± 0.7, respectively. Final yield was 6.2% ± 0.7 for CNC-CH and 4.2% ± 0.3 for CNC-EN.

### 2.2. CNC Characterization

**Figure 1:**
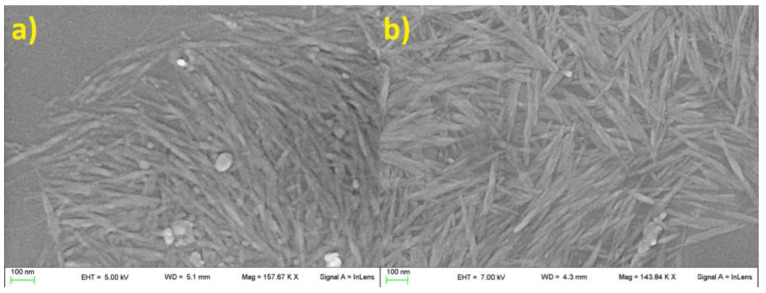
CNC-CH (**a**), CNC-EN (**b**), observed through SEM. No particular differences in size and shape were observed, other than the presence of a larger mass of impurities in CNC-CH samples.

**Figure 2:**
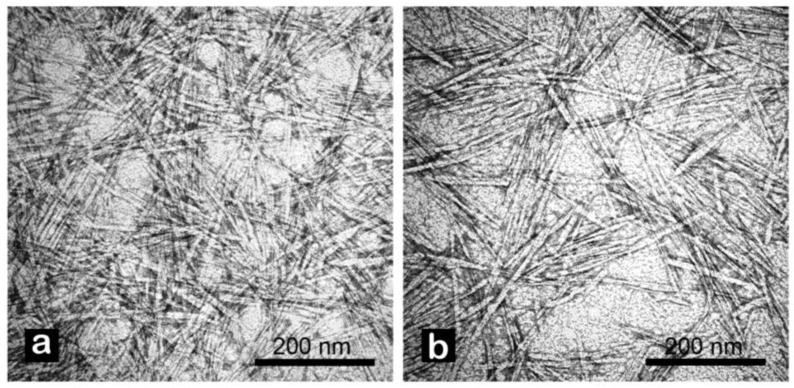
TEM analysis of CNC from tomato waste obtained by chemical (**a**), and enzymatic (**b**) extraction protocols. Images were recorded at 100 kX. Samples were stained with 2% uranyl acetate in distilled water prior to observation.

Morphology of tomato CNC samples was not affected by the two extraction protocols. In both cases, samples were characterized by the classical acicular shape with an apparent length between 100 and 300 nm (Figure 1 and Figure 2). A higher presence of aggregated matter was observed in CNC-CH (Figure 1a). The aggregation increased with dilution, suggesting potential hydrophobic or low-soluble material that precipitated by increasing the water amount.

**Figure 3:**
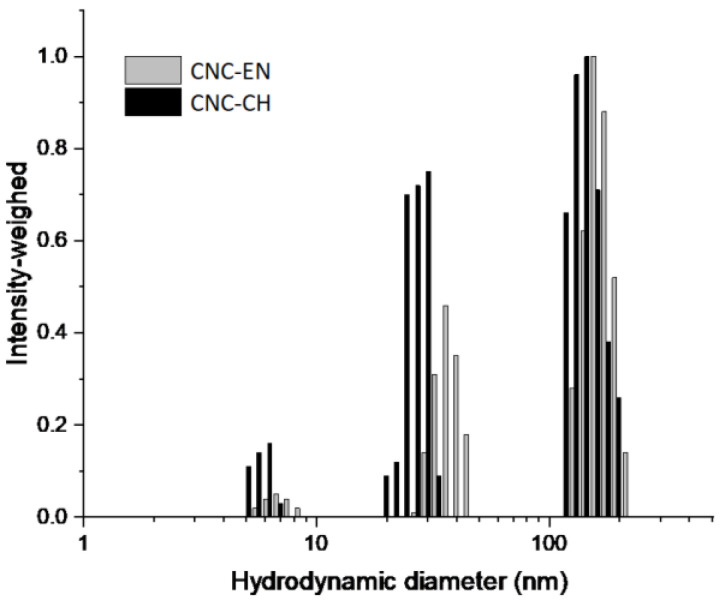
Hydrodynamic size distributions of tomato CNC-CH and CNC-EN. Measurements were performed at r.t. in water. The populations below 10 nm were ascribed to process residual impurities.

Particle size analysis confirmed the morphological observations. Both conditions produced CNC populations with a Nicomp mean hydrodynamic diameters (NMHD) of around 150 nm and smaller populations of around 30–40 nm and 7 nm (Figure 3). These populations are potentially ascribable to impurities arising from process residues. Consistently with SEM analysis, these smaller populations produced a higher scattering intensity in the chemically extracted samples (Figure 3) in line with the postulated higher contamination of the CNC-CH compared with CNC-EN.

**Figure 4:**
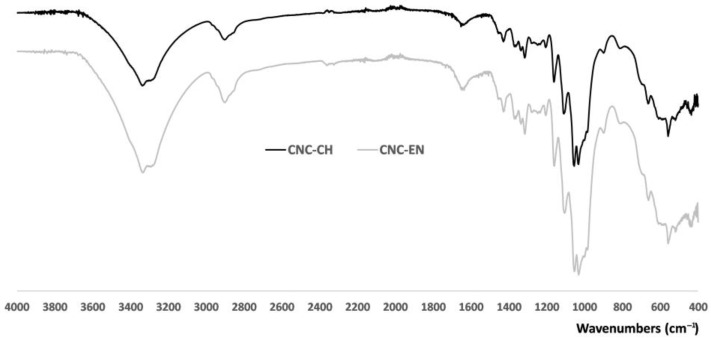
ATR-IR spectra of CNC-CH and CNC-EN. Unprocessed powder samples were analyzed directly at r.t. in the 400–4000 cm^−1^ region, acquiring100 scans with a resolution of 4 cm^−1^.

ATR-IR analysis showed similar absorption bands comparable with those of microcrystalline cellulose (Figure S1), which indicates that there was no new formation of bonds during acid hydrolysis or enzymatic extraction (Figure 4). The large band of CNC at 3500–3300 cm^−1^ corresponds to O-H stretching, including intermolecular hydrogen bonds, while the band around 2900 cm^−1^ is due to the C-H and CH_2_ stretchings. In the highly characteristic fingerprint region of the two spectra, the peaks at 1429, 1313, 1032, and 898 cm^−1^ represent typical cellulose absorption peaks. Each peak can be assigned, respectively, to CH_2_ bending, CH_2_ rocking, C-O stretching and C-H bending [26]. The spectral band at 898 cm^−1^ is also associated with the cellulosic β-glycosidic bonds (group C1 frequency) [27]. The presence of this absorption band and the absence of a peak at 893 cm^−1^ further corroborate the XRD results. Owing to the absence of the 893 cm^−1^ peak that is characteristically assigned to the frequency of the C1 group in cellulose II, we can argue that the CNC type II is almost negligible in the extracts [28].

**Figure 5:**
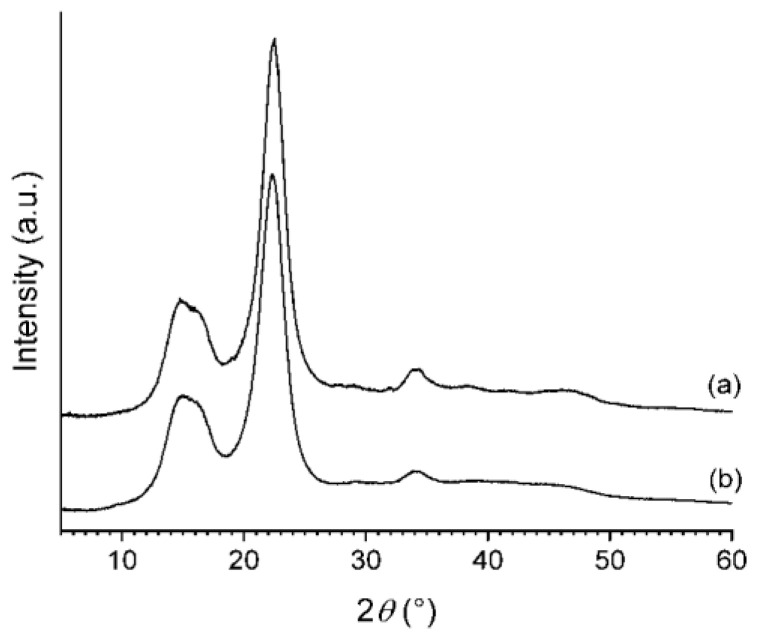
XRD patterns of CNC-EN (**a**), and CNC-CH (**b**).

**Figure 6:**
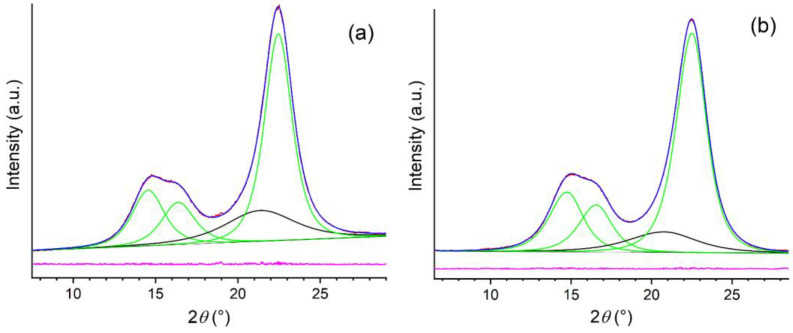
Profile fits of XRD patterns of CNC-EN (**a**), and CNC-CH (**b**), in which the individual diffraction peaks of cellulose Iβ are in green, the contribution of the amorphous fraction is in black, the experimental pattern is in red, the global profile fit is blue, and their difference is in pink.

XRD patterns of the freeze-dried CNC-CH and CNC-EN are very similar (Figure 5); the phase analysis showed the presence of only one crystalline form in both samples: cellulose I. This form is known to exist in two modifications, α and β; however, their peak positions and intensities are close to one another, so they cannot be separately quantified due to the large broadening of the experimental patterns. Therefore, the patterns were decomposed assuming the presence of only the most common cellulose Iβ. The refinement procedure provided very similar degrees of crystallinity: 81% for the CNC-EN and 79% for CNC-CH. Figure 6 shows the results of the profile fit procedure. The large broadening of diffraction peaks is also very similar for the two samples, around 2–2.5° 2θ in terms of full width at half maximum. The application of the Scherrer equation to these values suggests an average coherent crystalline domain size of about 3–4 nm.

### 2.3. Antimicrobial Properties of CNC

**Figure 7:**
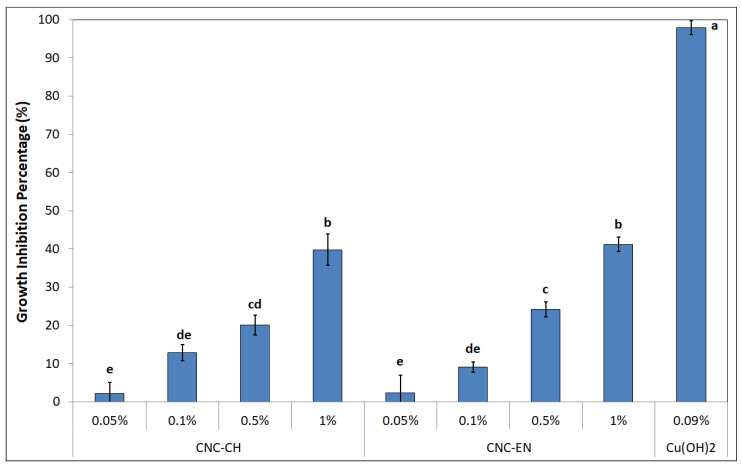
Growth inhibition percentage showed by CNC-CH and CNC-EN on Pto after 24 h. Data represent the mean and standard deviation; different letters show significantly different values after one-way ANOVA, followed by Tukey’s HSD post hoc test (*p < 0.05*).

Both CNC were tested for their antimicrobial properties on the model plant pathogen Pto. Albeit far from the nearly 100% inhibition of copper hydroxide, both CNC-CH or CNC-EN showed a dose-dependent effect on bacterial growth after 24 h, with CNC-CH and CNC-EN achieving 39.8 and 41.2% inhibition, respectively, at 1% *w/v* (Figure 7).

**Figure 8:**
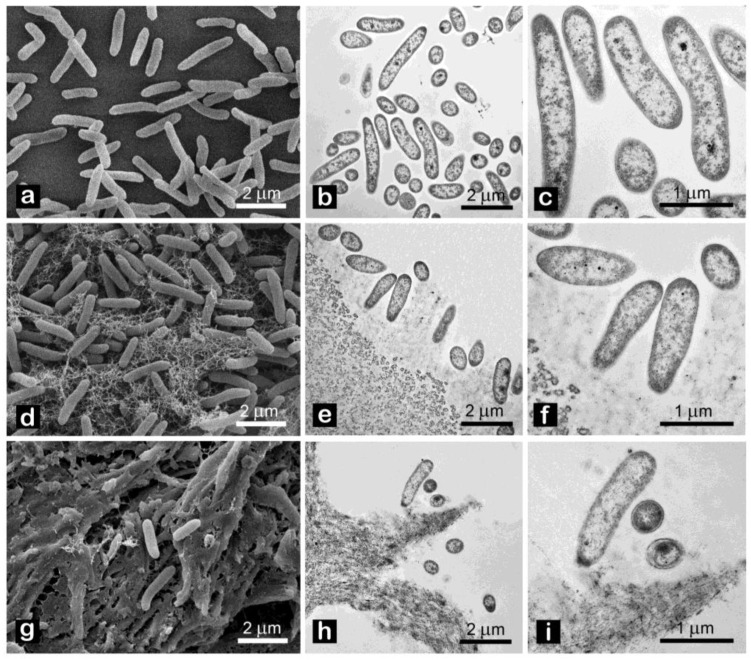
Pto morphology after exposure for 12 h to: tap water ((**a**) SEM, (**b**,**c**) TEM); 1% *w/v* CNC-CH (**d**) SEM, (**e**,**f**) TEM); 1% *w/v* CNC-EN (**g**) SEM, (**h**,**i**) TEM).

Effects of CNC on cell morphology were investigated by electron microscopy. Both SEM and TEM analyses revealed no peculiar changes, such as loss of membrane integrity, vacuolization or presence of vesicles, of cell ultrastructure after 12 h exposure (Figure 8).

**Figure 9:**
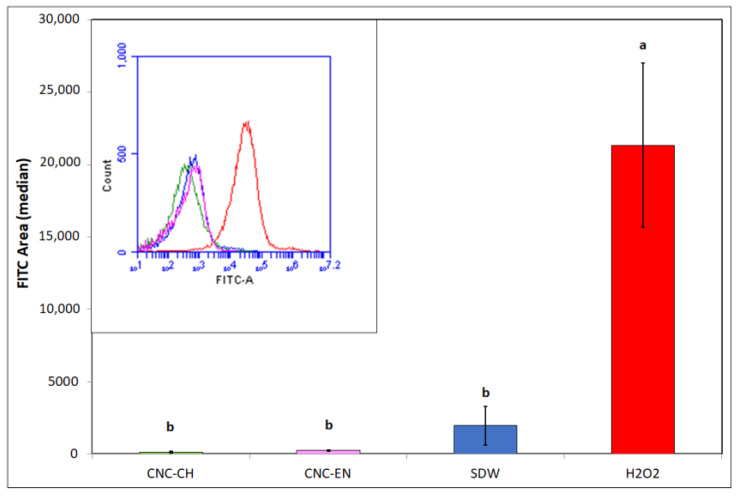
ROS production in Pto cells with exposition to 1% *w/v* CNC-CH, 1% *w/v* CNC-EN and 2% *v/v* H_2_O_2_ after 12 h. Data represent the mean and standard deviation; different letters show significantly different values after one-way ANOVA, followed by Tukey’s HSD post hoc test (*p* < 0.05).

Measurements of intracellular ROS by DCFH-DA cytofluorimetric readings clearly showed that CNC did not generate oxidative stress in the bacterial cells, since the obtained values after 12 h were comparable to the negative control (sterile deionized water—SDW), while the positive control (H_2_O_2_) was ten-fold higher (Figure 9).

**Figure 10:**
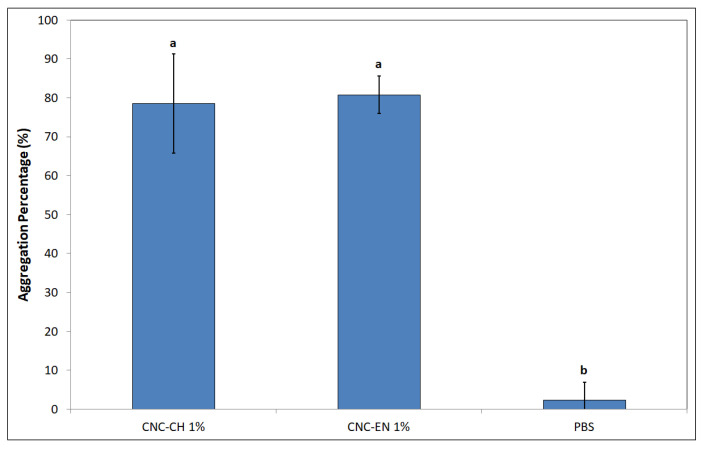
Aggregation of Pto cells after 12 h exposition to 1% *w/v* CNC-CH and 1% *w/v* CNC-EN. Data represent the mean and standard deviation; different letters show significantly different values after one-way ANOVA, followed by Tukey’s HSD post hoc test (*p* < 0.05).

The jar coagulation test revealed a very different behavior of bacterial cells when exposed to a suspension of 1% *w/v* CNC (Figure 10). After 3 h, aggregation level was equal to 78.5 and 80.8% for CNC-CH and CNC-EN, respectively, compared to the almost not aggregated control (phosphate buffer saline—PBS).

**Figure 11:**
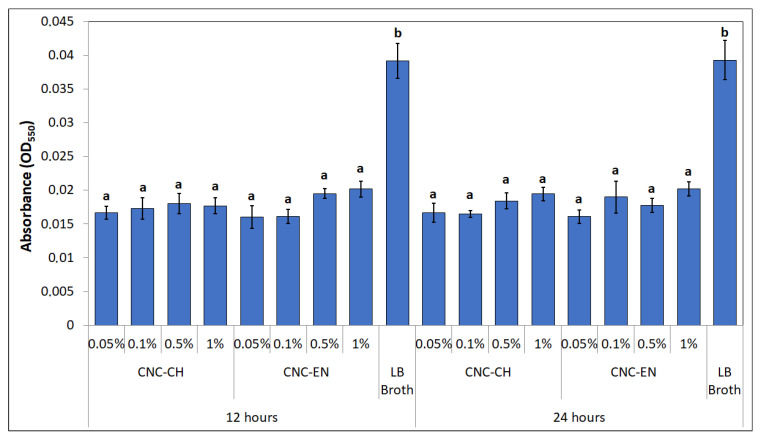
Biofilm production at 12 and 24 h. Data represent the mean and standard deviation; different letters show significantly different values after one-way ANOVA, followed by Tukey’s HSD post hoc test (*p* < 0.05).

Interesting results were obtained by studying the produced biofilm of Pto in the presence of CNC. At 12 and 24 h of incubation, biofilm-related absorbance values were statistically lower than control (LB broth alone). Notably, the biofilm was inhibited even at concentrations ineffective on bacterial growth (Figure 11).

**Figure 12:**
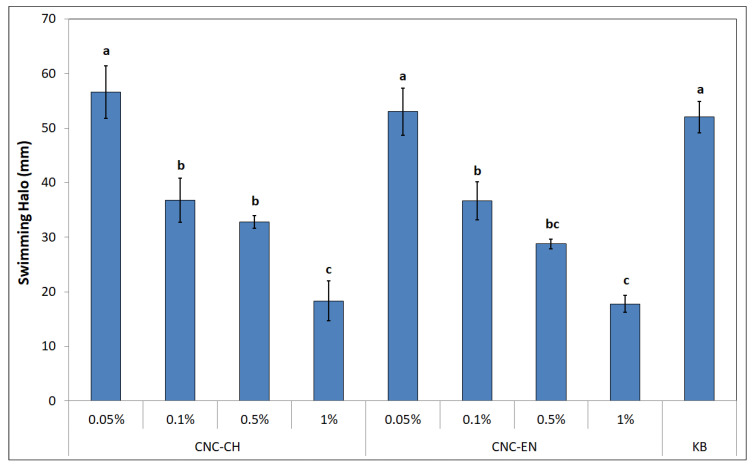
Swimming halos of Pto after 48 h of incubation at 27 °C on soft agar amended with different concentrations of CNC. Data represent the mean and standard deviation; different letters show significantly different values after one-way ANOVA, followed by Tukey’s HSD post hoc test (*p* < 0.05).

CNC seem to influence bacterial motility, as well. After 48 h of incubation on soft KB (0.3% *w/v* agar), different swimming halos were recorded with different CNC concentrations (Figure 12). The best results were obtained with 1% CNC-CH and CNC-EN, with 18.3 and 17.7 mm halos, which correspond to a swimming inhibition of 64.7 and 65.8%, respectively. Even lower concentrations of CNC (0.5 and 0.1%) displayed good results ranging from 28 to 36 mm, suggesting a dose-dependent effect, while the lowest tested concentration of 0.05% was not statistically different from the control (KB alone).

## 3. Discussion

Tomato post-harvest residues are emerging as promising low-cost sources of cellulose. Both the proposed protocols, chemical-based and enzymatic digestion-based, were able to isolate cellulose at acceptable yields and could subsequently be shaped into a crystalline nanoform by acid hydrolysis. Although cellulose represents a renewable and affordable polymer, the conventional process of extraction from lignocellulosic biomasses requires a huge amount of chemicals, the use of which has led to a questioning of the sustainability of the whole procedure at an industrial level, pushing the research towards greener extraction methods able to preserve its properties [29]. In this work, we demonstrated the possibility of performing one single bleaching treatment with an inferior total amount of NaClO_2_ (1.7–1.8 fold lower) compared to that used in similar works [21,30]. Moreover, we were able to avoid the use of NaHSO_4_ and to lower the final amount of used HCl for CNC synthesis, from 64% to 62%, by submitting the tomato fibers to enzymatic digestion by a mix of cellulases, β-glucosidases and hemicellulases. This short enzymatically driven depolymerization was conducted for 24 h, to lower the amount of reducing sugars belonging to the amorphous regions of cellulose, and to obtain a residue richer in crystalline cellulose. Comparing the final amount of fibers obtained by the two protocols, the addition of enzymatic digestion led to a drastic reduction in cellulose yield, from 28 to 13%. Despite this, the quality of cellulose increased, and thus it was possible to lower the concentration of the acid hydrolysis while increasing its final yield (31.3%). CNC-CH and CNC-EN presented the same morphology and dimensions (acicular, rod-shaped crystals with length around 150 nm), but the latter had a smaller amount of impurities and a slightly, although no significantly, higher crystallinity index (81%). To increase the level of CNC purity, the following methods can be pursued: grinding with a blender, deep grinding with a colloidal mill, and atomization using a spray-dryer [31]. The increased hydrolysis yield and crystallinity index, as well as lower impurities, could be explained considering a cleaner starting bulk of cellulose, obtained thanks to the enzymatic treatment, that was able to eliminate most of the amorphous portions and hemicelluloses in the fibrils, enhancing acid hydrolysis. No differences were recorded among CNC-CH and CNC-EN in terms of composition, as displayed by XRD and ATR-IR analysis, supporting the introduction of an enzymatic digestion step in the process of CNC synthesis. While some authors have already pointed out the potential of applying different, less chemical-based protocols to obtain cellulose from tomato waste, more experiments need to be performed to achieve the best combination of treatments (chemical, mechanical, enzymatic) in terms of final yield and environmental impact, as well as industrial scalability. The final yield displayed by the enzymatically driven protocol was lower (4.2%), even if not in a statistically significant way, compared to the chemical one (6.2%). In this sense, further insight could be acquired by investigating the effects of different enzymatic concentrations by varying at the same time process parameters, such as time and temperature.

Regarding CNC antimicrobial activity on Pto cells, no differences were recorded between CNC-CH and CNC-EN, indicating that the proposed synthesis processes do not affect their effect on microorganisms. CNC have been shown to possess the ability to weakly inhibit bacterial growth at high concentrations (1% *w/v*), although far from the efficacy of cupric compounds traditionally used in pest management. Similar results have been recorded on different bacterial pathogens. Noronha et al. (2021) demonstrated the ability of CNC to inhibit 90% of *E. coli* cells, suggesting the disruption of membrane integrity as one of the main antimicrobial mechanisms, deduced by investigating the CNC effect on artificial lipid vesicles [22]. Focusing on bacterial plant pathogens, different levels of inhibition by CNC have been recorded on *Xanthomonas arboricola* pv. *corylina* (Xac) and *Pseudomonas savastanoi* pv. *savastaonoi* (Psav), suggesting that effects on growth inhibition are bacterial species-dependent [32,33]. Our results indicate that CNC exposure does not lead to shape modification or other appreciable ultrastructural changes in Pto cells, as confirmed by electron microscopy investigations, excluding a direct interaction with the bacterial membranes or internalization of the nanomaterials. This was also confirmed by the absence of detected intracellular ROS, which have been observed in many previous studies with nanomaterials and microorganisms [34,35]. In the proposed experiment, DCFH-DA, after being absorbed by the cell and hydrolyzed in DCFH, reacts with intracellular ROS to produce highly fluorescent dichlorofluorescein, whose intensity is proportional to the amount of ROS [36]. The detected fluorescence was statistically comparable to the negative control; hence, we can exclude that CNC induce oxidative stress on bacterial cells. The antimicrobial properties of CNC could depend more on their impact on bacteria-environment interactions. Similarly to what has been reported by other authors, CNC could lead to the aggregation and flocculation of the bacterial cells in a liquid medium [37,38]. After several hours of exposure, a huge aggregation was recorded for both tested CNC samples. CNC negative surface charges as well as the suspension pH have been considered the main factors responsible for the depletion of bacteria interaction [39]. This phenomenon could also play a major role in preventing and disrupting bacterial ability to adhere to surfaces, explaining why a significant biofilm inhibition was observed in Pto cells treated with CNC after 12 and 24 h, even at those concentrations that showed limited growth inhibition. A similar behavior has already been described on Psav [33]. Furthermore, cellulose nanofibers (CNF) have been shown to reduce biofilm production with the foodborne bacteria *L. monocytogenes* [40]. Since attachment to surfaces plays a fundamental role in early biofilm formation, preventing bacterial adhesion could explain the low mass of biofilm measured [41]. Indeed, the ability of CNC to prevent cell adhesion has already been pointed out on *E. coli* [22,42]. Another interesting effect emerged from our experiments is the ability of CNC to diminish bacterial swimming. Swimming in flagellated bacteria is considered one of the main mechanisms for motility in liquid media, and is a major virulence trait in plant pathogenic bacteria [43,44]. Similarly, CNF have been recorded to reduce swimming motility in several foodborne bacterial pathogens, such as *L. monocytogenes*, *E. coli* and *B. cereus*, while Sakata et al. (2022) highlighted the potential of covering cabbage leaves with CNF to prevent *Pseudomonas cannabina* pv. *alisalensis* to move inside the plant through swimming, pointing out a downregulation of bacterial flagellin gene *fliC* [40,45]. To the best of our knowledge, this is the first observation of bacterial swimming inhibition by CNC. Since many complex interactions occur in bacterial motility-to-biofilm transition, a multiple contribution from each of the cited antimicrobial mechanisms may explain the observed antibacterial properties on Pto [46].

Further investigations are ongoing on the effect of CNC in artificially inoculated plants. Our results give for the first time a much more comprehensive insight into the antimicrobial properties of CNC, suggesting their potential role in controlling plant pathogenic bacteria by inhibiting those traits involved in epiphytic colonization. Understanding how to maximize these properties could lead to the development of innovative lignocellulosic nanoagrochemicals in a circular economy context [47].

## 4. Materials and Methods

All the cited chemicals were supplied by Sigma-Aldrich, Inc. (Taufkirchen, Germany), and used without further modifications.

### 4.1. Cellulose Extraction and CNC Synthesis from Tomato Waste

Tomato harvest residues were collected from fields in Monticelli d’Ongina (PC, Italy) at the end of summer 2021. Stems, petioles and leaves were washed in deionized water to remove soil debris, then dried at 40 °C for 24 h and finally grinded.

#### 4.1.1. Chemical Extraction Protocol and Synthesis

The following chemical protocol was proposed to isolate cellulose and synthesize nanocrystals (CNC-CH): 30 g of raw lignocellulosic powder were autoclaved in deionized water at 140 °C for 30′ at 2 atm. The pulp was then treated twice with 5% *w/v* NaOH at 80 °C for 2 h and dried at 60 °C for 24 h. The biomass was bleached with pH 3.5 3% *w/v* NaClO_2_ at 98 °C for 2 h, and then with 5% *w/v* NaHSO_4_ at room temperature for 30′. The final biomass was dried at 60 °C until constant weight was reached. Yield of the extraction process was calculated as percentage ratio between the final and the initial weight of the sample. The obtained cellulose was then hydrolyzed under continuous stirring using a 64% *v/v* HCl solution at 45 °C for 30′. The resulting suspension was dialyzed against deionized water until pH 6.5 was reached. Then, the suspension was ultrasonicated using a dip-probe at 700 W for 8′. The final aqueous suspension was stored at 4 °C until analysis. The acid hydrolysis yield was calculated using the standard method UNI EN ISO 638:2009 [10]. Final yield of the whole process was calculated by multiplying the cellulose extraction yield by the acid hydrolysis yield. Each extraction and synthesis was repeated three times.

#### 4.1.2. Enzymatic Extraction Protocol and Synthesis

A modified extraction protocol was also investigated by introducing an enzymatic digestion step, and the properties of the obtained nanocrystals (CNC-EN) were compared with CNC-CH. The conventional protocol was modified as follows: NaClO_2_ bleaching was reduced at 1.5% *w/v* but repeated twice instead of performing the NaHSO_4_ treatment. After drying, 5 g of the biomass was incubated in 50 mL of 50 mM sodium citrate buffer with a dosage of 10% *w/w* enzyme/dried pulp. The enzymatic cocktail used was a blend of cellulases, β-glucosidases and hemicellulases (Cellic, Ctec2) [48]. The process was conducted for 24 h at 50 °C at 100 rpm. The enzymatically treated matrices were then filtered, washed to reduce excess buffer, and dried at 60 °C for 24 h. Cellulose extraction yield was calculated before and after enzymatic digestion. CNC were obtained as already described, but lowering the concentration of the acidic solution from 64 to 62% *v/v*. As above, the acid hydrolysis yield was calculated using the standard method UNI EN ISO 638:2009. Final yield of the whole process was calculated multiplying the cellulose extraction yield by the acid hydrolysis yield. Each extraction and synthesis was repeated three times.

### 4.2. CNC Characterization

#### 4.2.1. IR Spectroscopy

The FTIR-ATR spectra (internal reflection accessory) were recorded using an IR Spirit Infrared Spectrophotometer (Shimadzu, Kyoto, Japan) in the spectral range of 400–4000 cm^−1^ and with a resolution of 4 cm^−1^ at 100 scans. A small quantity of the dried powdered samples was deposited directly on the ATR crystal plate with no sample processing. Background spectrum was acquired on the empty crystal plate.

#### 4.2.2. X-ray Diffraction

X-ray powder diffraction (XRD) patterns were collected with a D8 Advance diffractometer (Bruker AXS GmbH, Karlsruhe, Germany) in Bragg–Brentano geometry, equipped with a Lynxeye XE-T position sensitive detector, using the Cu-Kα radiation (40 kV, 40 mA). Data were collected in the 4–60° 2θ range using a 0.030° step scan and 1 s counting time. The patterns were decomposed in the contributions of cellulose Iβ reflections, and the amorphous content by means of a profile fit procedure with pseudo-Voigt functions, in the 7–29° 2θ range, using the Philips ProFit software, following the method described by C. J. Garvey et al. (2005) [49,50]. The crystallinity index was estimated as the wt% of the crystalline phase, assuming that the area under the diffraction peaks was proportional to the weight amount of each phase.

#### 4.2.3. Size and Morphology Analysis

Photocorrelation spectroscopy was employed to investigate CNC size distribution in samples obtained by chemical and enzymatic extraction processes. Samples were analyzed directly in the extraction solution using a Nicomp 380 ZLS photocorrelator (PSS, Santa Barbara, CA, USA) equipped with a 55 mW He-Ne Coherent Innova 70–3 Laser source (λ = 654 nm) and APD detector. Samples were opportunely diluted with water and analyzed at 20 °C. Gaussian and Nicomp algorithms were employed for fitting the autocorrelation function time decay data. Analyses were run to convergence of the fitting algorithms, and the respective mean diameters were expressed as Gaussian and Nicomp mean hydrodynamic diameters (GMHD and NMHD, respectively).

CNC morphology was investigated by transmission electron microscopy (TEM) and scanning electron microscopy (SEM) using a Zeiss LEO 1525 field-emission microscope equipped with a GEMINI column (Oberkochen, Germany). For TEM analysis, droplets of CNC suspensions (10 µL) were placed on formvar carbon-coated grids and allowed to adsorb for 60 s. Excess liquid was removed gently by filter paper. The adsorbed specimen was then processed for negative staining, by first washing the specimen grid on a drop of negative stain (2% uranyl acetate in distilled water), blotting, and repeating this step leaving the specimen grid for 60 s on a new drop of negative staining solution. Samples were observed on a JEOL 1200 EX II electron microscope. Micrographs were acquired by the Olympus SIS VELETA CCD camera equipped with the iTEM software. Samples for SEM analysis were prepared by depositing properly diluted CNC suspensions onto a round glass specimen fixed on an aluminum specimen stub covered with a double-sided carbon adhesive disc. The sample was dried at room temperature and coated with chromium before imaging for 20 s at 20 mA (Quorum Q150T ES East Grinstead, West Sussex, UK).

### 4.3. Antimicrobial Properties of CNC

#### 4.3.1. Bacterial Growth Inhibition

To investigate the potential antimicrobial mechanisms of CNC, the model plant pathogen *Pseudomonas syringae* pv. *tomato* (Pto) strain DC3000 was cultivated in vitro on King’s B medium (KB) for 48 h at 27 °C [51]. Effects on the bacterial growth were tested by making CNC suspension in sterilized tap water at different concentrations (0.05, 0.1, 0.5 and 1% *w/v*). To help CNC’s dispersion, the suspensions were bath-sonicated at 20 kHz for 20 min [52]. Tap water alone and with copper hydroxide at 0.09% *w/v* was used as control [53]. Each suspension was combined with an aliquot of Pto cells harvested from a fresh plate, reaching a final concentration of 10^3^ CFU mL^−1^. Then, 2 mL of each suspension was incubated in tubes under continuous orbital shaking at 27 °C. After 24 h for each tube, several dilutions were made, and 100 µL of each dilution was plated on KB petri dishes. Formed colonies were counted after 48 h of incubation at 27 °C. For each sample, three replicates were made, and for each dilution, three plates were made. The experiment was repeated twice. Growth inhibition is expressed as a percentage by applying the following formula:

(1) $$Growth\;Inhibition=\frac{Water\;control\;Colonies-Sample\;Colonies}{Water\;control\;Colonies}\times 100\;$$

#### 4.3.2. Cell Observation

Effects of CNC on the morphology of bacterial cells were investigated by electron microscopy. Suspensions of 10^7^ CFU mL^−1^ Pto cells were obtained in tap water tubes containing 1% *w/v* of CNC. Sterile tap water served as control. After 12 h of incubation under continuous shaking at 27 °C, tubes were centrifuged, supernatant discarded, and the obtained pellet was washed twice with phosphate buffer saline (PBS), before being processed as follows. For scanning electron microscopy, samples were pre-fixed for 30 min at 4 °C with 2.5% glutaraldehyde in cacodylate buffer 0.1 M pH 7.3 containing 0.075% ruthenium red and 0.075 M lysine acetate. After washing three times for 10 min each at 4 °C in the same buffer, they were fixed with 2.5% glutaraldehyde in cacodylate buffer 0.1 M pH 7.3 for 2 h at 4 °C. Samples were washed again, three times for 10 min each at 4 °C in the same buffer, and were post-fixed with 2% osmium tetroxide in cacodylate buffer for 2 h at 4 °C. Specimens were washed in the same buffer, three times for 15 min each at 4 °C and dehydrated in a graded ethanol series. Samples were dried by the critical point method using CO_2_ in a Balzers Union CPD 020. They were attached to aluminum stubs using a carbon tape and sputter-coated with gold in a Balzers MED 010 unit. The observation was made using a JEOL JSM 6010LA electron microscope. For transmission electron microscopy, samples were fixed and dehydrated as described above and then infiltrated with mixtures of Epon resin/ethanol in different percentages. At the end of the procedure, samples were embedded in pure resin and let to polymerize for 2 days at 60 °C. Blocks were cut with Reichert Ultracut ultramicrotome using a diamond knife. For TEM, ultrathin sections (60–80 nm) were collected on copper grids, stained with uranyl acetate and lead citrate, and observed with a JEOL 1200 EX II electron microscope. Micrographs were captured by the Olympus SIS VELETA CCD camera equipped with iTEM software.

#### 4.3.3. Measurements of Intracellular ROS

To evaluate generation of ROS in Pto cells, 2,7-dichlorofluorescein diacetate (DCFH-DA) was used in this experiment. Bacterial concentration of 10^7^ CFU mL^−1^ was achieved in 1% *w/v* CNC suspensions made in tap water. Water and 2% *v/v* H_2_O_2_ were used as controls. Tubes were incubated as described in Section 4.3.2, then 100 µM DCFH-DA was added and allowed to react in the dark for 1 h. ROS were detected at 485/20 nm of fluorescence excitation wavelength and 528/20 nm of emission wavelength using a cytofluorimeter (Accuri C6 Plus, BD Bioscences, Franklin Lakes, NJ-USA) [54]. Values (presented as the average median of the fluorescent intensity in the FITC region) were obtained from the reading of 15,000 events per sample. Each thesis consisted of three replicates.

#### 4.3.4. Jar Coagulation Assay

To calculate the effect of CNC on the aggregation of cells, the OD_600_ of a Pto suspension was measured [37]. A concentration of 10^7^ CFU mL^−1^ was achieved in 1 mL of a 1% *w/v* CNC suspension made using PBS. Tubes were statically incubated for 3 h, then they were centrifuged at 600 rpm for 90 s. The supernatant was transferred, and its absorbance was measured. PBS alone was used as control. Suspensions without bacteria were used as blanks. Aggregation percentage was calculated as follows, after subtracting the blank to the samples:

(2) $$Aggregation=\frac{OD600\;Control-OD600\;Sample}{OD600\;Control}\times 100\;$$

The experiment was repeated twice. Each experiment consisted of three replicates.

#### 4.3.5. Biofilm Production

The effects of CNC on bacterial biofilm production were observed through a microplate experiment as previously described by O’Toole (2011), with few adjustments [55]. Briefly, a final volume of 100 µL was achieved in each well of a 96-well microplate, by adding to 90 µL of a CNC suspension made in Luria-Bertani broth (LB), according to Section 4.3.1., 10 µL of a Pto suspension, reaching a final concentration of 10^6^ CFU mL^−1^. Plates were statically incubated at 27 °C. After 12 and 24 h, plates were gently washed twice in deionized water and 100 µL of 0.1% *w/v* crystal violet were poured in each well. After a short incubation for 20 min at room temperatures, plates were rinsed thrice in water and let dry overnight. After that, 125 µL of 30% *v/v* acetic acid was poured into each well. Their contents were transferred to a new plate after an incubation of 20 min at room temperature; then, biofilm was measured using a spectrophotometer set at 550 nm (Diatek Instruments). LB broth alone was used as control. Acetic acid alone was used as blank. For each thesis 8 replicates were taken. The experiment was repeated twice.

#### 4.3.6. Swimming Motility

To assess the role of CNC in bacterial swimming motility, 0.3% agar KB plates were supplemented with CNC to reach the final concentration indicated in Section 4.3.1 [56]. A 5 µL volume of 10^6^ CFU mL^−1^ Pto suspension was pipetted into the center of each plate, and was then incubated for 48 h at 27 °C. KB alone was used as control. After incubation, the swimming activity was quantified by measuring the diameter of the visible halo. Data were obtained from two experiments, each of which consisted of three replicates per thesis.

### 4.4. Statystical Analysis

Data from the experiments were subjected to one-way analysis of variance (ANOVA). Two levels of significance (*p*  <  0.05 and *p*  <  0.01) were computed to assess the significance of the F values. For each average value, the standard deviation (±SD) was calculated. A pairwise analysis was carried out using Tukey’s HSD test at confidence levels of 0.95 or 0.99. Statistical analyses were performed using XLSTAT 2020.4 (Addinsoft, Paris, France).

## 5. Conclusions

Valorization of lignocellulosic waste is a constant challenge in agriculture. In this work, we showed that tomato residues can be successfully used as a renewable source of CNC by more sustainable chemical and enzymatic approaches, thus leading to a decrease in chemicals traditionally used in the synthesis process. More notably, both protocols produced CNC with similar properties, as well as promising antimicrobial activity against plant bacterial pathogens, suggesting a potential role of these nanomaterials as novel compounds to control bacterial plant pathogens.

## Data Availability

Not applicable here.

## Supplementary Materials

The following supporting information can be downloaded at: https://www.mdpi.com/article/10.3390/plants12040939/s1, Figure S1: ATR-IR spectra of CNC and CMC.
