# Supplementary material for: Sustainable Protocols for Cellulose Nanocrystals Synthesis from Tomato Waste and Their Antimicrobial Properties against Pseudomonas syringae pv. tomato
Source: Plants (Basel). 2023 Feb 18;12(4):939. doi: 10.3390/plants12040939 (PMC9963933; doi:10.3390/plants12040939)
Supplement: Supplementary file 1 [file plants-12-00939-s001.zip › plants-2223482-supplementary.pdf]

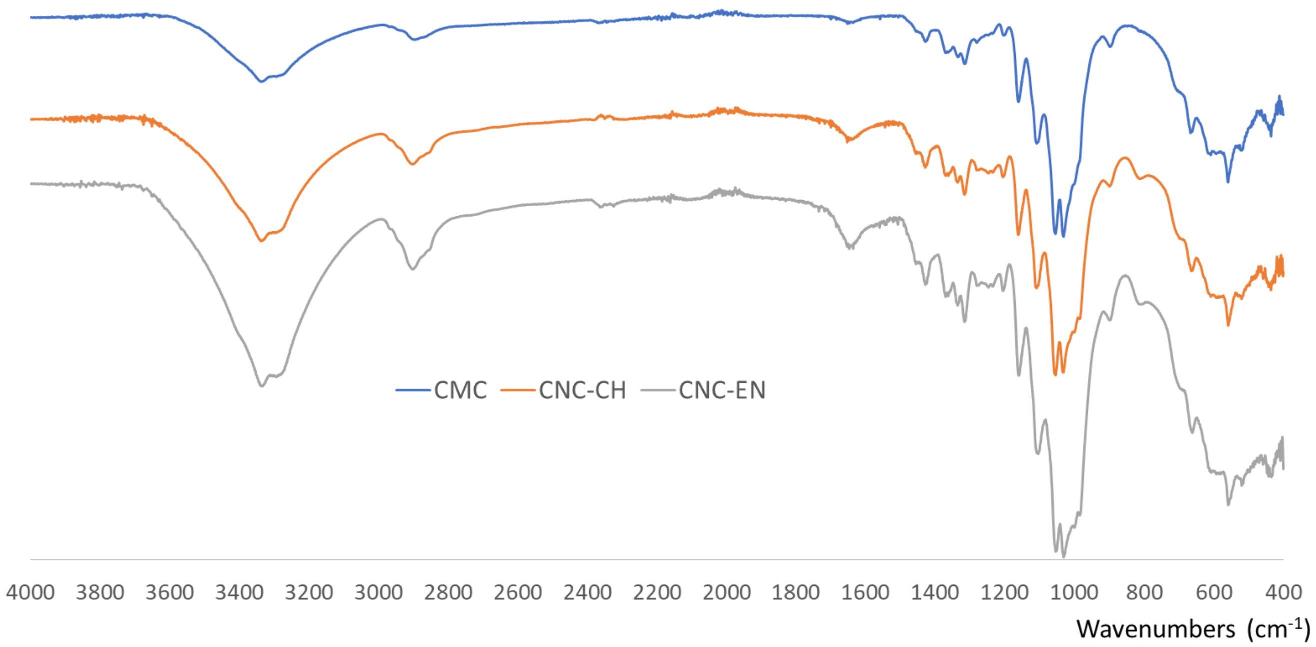

Figure S1. ATR-IR spectra of CNC-CH and CNC-EN in comparison with commercially available cellulose microcrystalline (CMC)(CMC for chromatography, CAS 9004-34-6, Sigma-Aldrich, Taufkirchen, Germany). Unprocessed powder samples were directly analyzed at room temperature in the 400-4000 cm^{-1} spectral region, acquiring 100 scans with a resolution of 4 cm^{-1} by using IRSpirit Infrared Spectrophotometer (Shimadzu, Japan).
